# Mechanistic insights into water adsorption and dissociation on amorphous 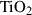-based catalysts

**DOI:** 10.1080/14686996.2017.1410055

**Published:** 2018-01-31

**Authors:** Kulbir Kaur Ghuman

**Affiliations:** ^a^ International Institute for Carbon Neutral Energy Research, Kyushu University, Fukuoka, Japan.

**Keywords:** Amorphous titanium dioxide, doping, catalyst, hydrogen, surface reaction, 50 Energy Materials, 205 Catalyst, Photocatalyst, Photosynthesis

## Abstract

Despite having defects, amorphous titanium dioxide ($\;{\text{aTiO}}_{2}$) have attracted significant scientific attention recently. Pristine, as well as various doped $\;{\text{aTiO}}_{2}$ catalysts, have been proposed as the potential photocatalysts for hydrogen production. Taking one step further, in this work, the author investigated the molecular and dissociative adsorption of water on the surfaces of pristine and $\;{\text{Fe}}^{2+}$ doped $\;{\text{aTiO}}_{2}$ catalysts by using density functional theory with Hubbard energy correction (DFT+U). The adsorption energy calculations indicate that even though there is a relatively higher spatial distance between the adsorbed water molecule and the $\;{\text{aTiO}}_{2}$ surface, pristine $\;{\text{aTiO}}_{2}$ surface is capable of anchoring $\;{\text{H}}_{2}\;\text{O}$ molecule more strongly than the doped $\;{\text{aTiO}}_{2}$ as well as the rutile (1 1 0) surface. Further, it was found that unlike water dissociation on crystalline $\;{\text{TiO}}_{2}$ surfaces, water on pristine $\;{\text{aTiO}}_{2}$ catalyst experience the dissociation barrier. However, this barrier reduces significantly when $\;{\text{aTiO}}_{2}$ is doped with $\;{\text{Fe}}^{2+}$, providing an alternative route for the development of an inexpensive and more abundant catalyst for water splitting.

## Graphical Abstract

Graphical abstract showing the reduction in water splitting barrier due to doping in amorphous $\;{\text{TiO}}_{2}$, bringing the catalytic acivity of amorphous $\;{\text{TiO}}_{2}$ close to crystalline $\;{\text{TiO}}_{2}$.

**Figure:**
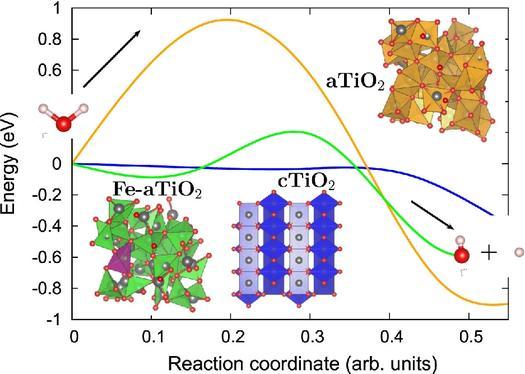

## 1. Introduction

Among various oxide semiconductor photocatalysts, crystalline forms of titanium dioxide (c$\;{\text{TiO}}_{2}$) have attracted significant attention in last decades as promising photocatalysts due to its biological and chemical inertness, strong oxidizing power and long-term stability against photocorrosion and chemical corrosion [1,2]. Where many crystalline oxide surfaces [3] including $\;{\text{cTiO}}_{2}$ surfaces [4–6] are extensively studied for their electronic structures, defect levels and polaron formation, amorphous $\;{\text{TiO}}_{2}$ ($\;{\text{aTiO}}_{2}$) despite having actual technical applications such as use as an active photocatalyst, a substrate, or a protection layer [7–9] waslacking many such investigations until recently. It is now well known that there is a possibility of using $\;{\text{aTiO}}_{2}$ as an inexpensive and more abundant alternative to $\;{\text{cTiO}}_{2}$ [10,11] which may lead to relatively simple and inexpensive technology in future.

**Figure 1.**
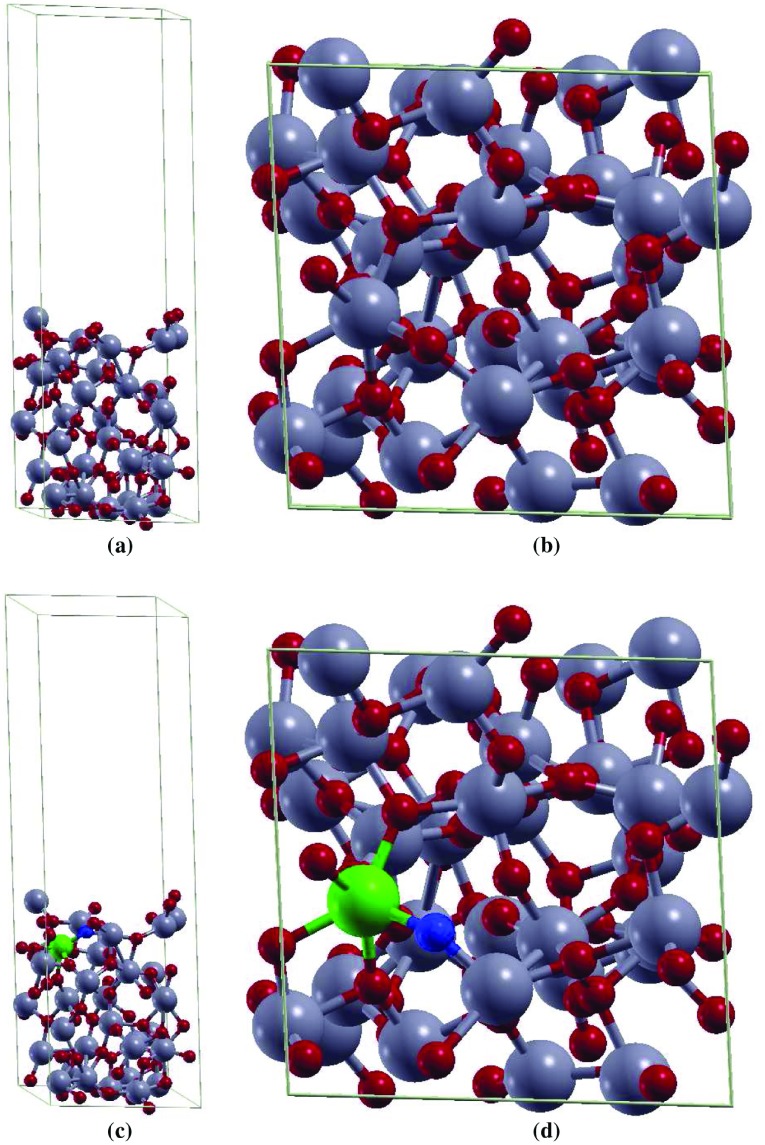
The (a) side and the (b) top view of 2×2×4 supercell of $\;{\text{aTiO}}_{2}$. The (c) side and the (d) top view of 2 × 2 × 4 supercell of Fe(II)-$\;{\text{aTiO}}_{2}$. Ti, O, Fe and O vacancy atoms are highlighted in grey, red, green, and blue, respectively.

**Figure 2.**
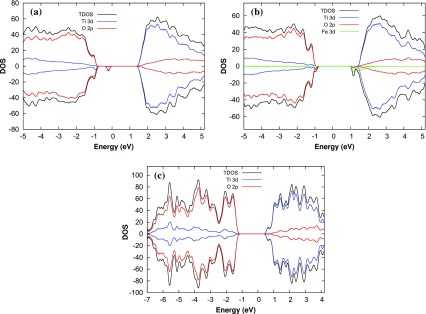
Total density of states and the projected density of states on the p and d orbitals of (a) $\;{\text{aTiO}}_{2}$, (b) Fe(II)-$\;{\text{aTiO}}_{2}$ , and (c) rutile (1 1 0) surface models.

Despite having qualitatively similar electronic structure as of $\;{\text{cTiO}}_{2}$ [10,11], $\;{\text{aTiO}}_{2}$ forms large localized bound state on several O and Ti atoms due to the strong electron interaction with the lattice distortion [10,12,13]. These localized states in $\;{\text{aTiO}}_{2}$ leads to self-trapping of holes, excess electrons and excitons which play important roles in the radiation-induced processes [14–16] such as water splitting. Moreover, very recentlymany studies showed that the photoactivity of $\;{\text{aTiO}}_{2}$ can further be enhanced by doping it with suitable dopants [17–23]. The synergetic role that the amorphousness and dopants play in the photoactivity of $\;{\text{aTiO}}_{2}$ was attributed to the unique position of the midgap states, high self-trap energy, low mobility and weak chemical bonds of doped $\;{\text{aTiO}}_{2}$ [24].

Inspite of these numerous studies proposing $\;{\text{aTiO}}_{2}$-based catalysts for water splitting, the fundamental insights into the reaction pathway for water dissociation on $\;{\text{aTiO}}_{2}$-based catalysts are still lacking. This motivated the author to analyse the associative and dissociative water adsorption on pristine and doped $\;{\text{aTiO}}_{2}$ surfaces using first-principles based theoretical investigations. Since our recent study [24] clearly indicated that the visible light absorption of $\;{\text{aTiO}}_{2}$ can be improved by doping it with $\;{\text{Fe}}^{2+}$ due to its unique properties, the author have considered $\;{\text{Fe}}^{2+}$ doped $\;{\text{aTiO}}_{2}$ (Fe(II)-$\;{\text{aTiO}}_{2}$) as a model for doped $\;{\text{aTiO}}_{2}$ catalyst. Further in this work, the performance of $\;{\text{aTiO}}_{2}$-based catalysts is also compared with $\;{\text{cTiO}}_{2}$, by analysing water splitting on rutile (1 1 0) surface with the same level of theory.

**Figure 3.**
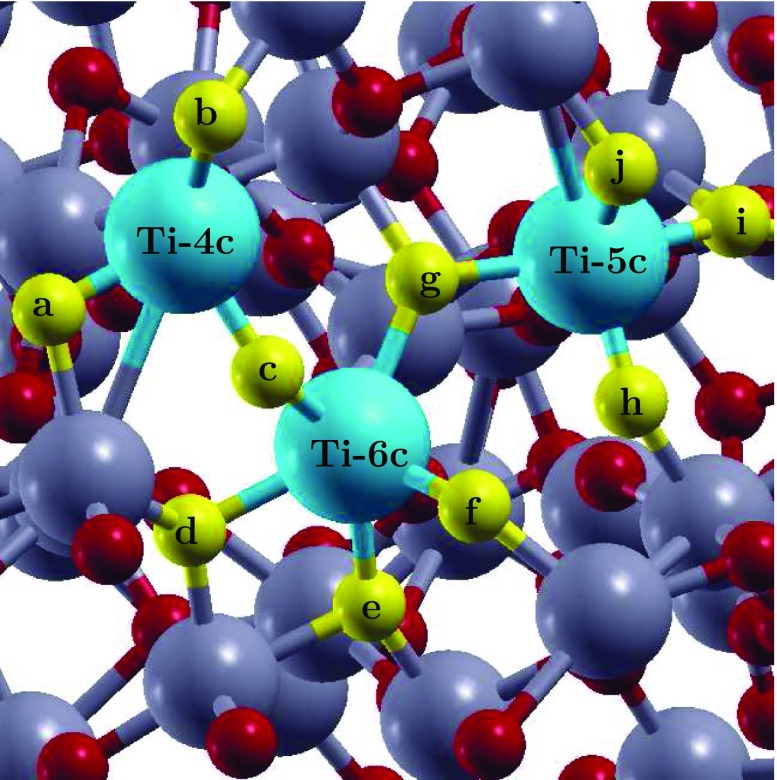
The $\;{\text{aTiO}}_{2}$ surface indicating the absorption sites for $\;{\text{H}}_{2}\;\text{O}$, OH and H species. Adsorption of $\;{\text{H}}_{2}\;\text{O}$ and OH was investigated by placing them on the top of the Ti atoms indicated in blue and adsorption of H was investigated by placing it on the top of oxygen atoms indicated in yellow. Rest of the Ti and O atoms are highlighted in grey and red, respectively.

## 2. Computational models and methods

The melt-quenching method was used to prepare $\;{\text{aTiO}}_{2}$ models [12]. The properties of the prepared $\;{\text{aTiO}}_{2}$-pagination models are in agreement with experimental and theoretical data available. The structural analysis of thesemodels suggests that local structural features of bulk $\;{\text{cTiO}}_{2}$ are retained in $\;{\text{aTiO}}_{2}$. In order to obtain the amorphous $\;{\text{TiO}}_{2}$ surface in the present study, a vacuum of about 20 Å  is added in the z direction of 2×2×4 bulk supercell (96-atom model) [25]. For doped $\;{\text{aTiO}}_{2}$ model, the author considered substitutional doping of a single Ti atom by one Fe atom and one O vacancy resulting into Fe(II) oxidation state with 3.125 at.% dopant concentration. The pristine $\;{\text{aTiO}}_{2}$ model and the doped $\;{\text{aTiO}}_{2}$ model indicating the positions of Fe atoms and O vacancy are shown in Figure 1. This doping level can be readily achieved for 3d transition metals in rutile $\;{\text{TiO}}_{2}$ [26,27].

The electronic characteristics of $\;{\text{aTiO}}_{2}$ sample has been investigated using *ab initio* method. The plane wave pseudopotential approach, together with the Perdew–Burke–Ernzerhof [28] exchange–correlation functional, and Vanderbilt ultrasoft pseudopotentials [29] was utilized throughout. The kinetic energy cut-offs of 544 and 5440 eV were used for the smooth part of the electronic wavefunctions and augmented electron density, respectively. The Quantum-ESPRESSO code, PWSCF package [30], was used to perform the calculations. All calculations are spin polarized. The structures were relaxed by using a conjugate gradient minimization algorithm until the magnitude of residual Hellman–Feynman force on each atom was less than $10^{-2}$ Ry/Bohr. In all electronic density of states (DOS) and projected density of states (PDOS) plots a conventional Gaussian smearing of 0.007 Ry was utilized.

Appreciable underestimation of band gap and delocalization of d and f electrons are well-known limitations of DFT. Therefore, density functional theory with Hubbard energy correction (DFT+*U*) formalism was used in this study with U=4.2 eV applied to Ti 3d electrons and U=6.00 eV applied to Fe 3d electrons for analysing electronic properties of pristine and doped $\;{\text{aTiO}}_{2}$. The value of *U* for Ti has been chosen not solely on the basis of band gap but also depending on the property of interest [31], which in the current study, is the photocatalytic behaviour of $\;{\text{TiO}}_{2}$ that in-turn depends upon the position of band gap states and their effect on the electronic structure [32]. This value of *U* for Ti is consistent with theoretical investigations by Morgan et al. [33], who calculated it by fitting the peak positions for surface oxygen vacancies to experimental X-ray photoelectron spectroscopy data. Further the value of U for Fe is taken from Ref. [34] which showed that the DOS of FeO show characteristics similar to experiments [35] with U=6.0 eV. Lastly, in order to evaluate the minimum energy pathways (MEPs) and transition states (TSs) for the water splitting reaction and estimate the relative activation energy barriers faced during dissociation of $\;{\text{H}}_{2}\;\text{O}$ molecule, non-spin-polorized climbing image nudged elastic-band (CI-NEB) DFT calculations with 7 images [36–38] were performed.

## 3. Results and discussion

First, the electronic properties of pristine $\;{\text{aTiO}}_{2}$ and Fe(II)-$\;{\text{aTiO}}_{2}$ surfaces were analysed and compared with the electronic properties of rutile (1 1 0) surface. Then the active sites for the adsorption of $\;{\text{H}}_{2}\;\text{O}$, OH, and H species on pristine $\;{\text{aTiO}}_{2}$ and Fe(II)-$\;{\text{aTiO}}_{2}$ surfaces were investigated followed by the study of reaction mechanism for water splitting on them. Water splitting on rutile (1 1 0) surface was also analysed for comparison. One water molecule per unit cell was used for all the calculations.

### Electronic properties of $\;{\text{aTiO}}_{2}$, Fe(II)-$\;{\text{aTiO}}_{2}$, rutile (1 1 0) surfaces

3.1.

To understand the catalytic properties of amorphous and crystalline surfaces, the author first studied the electronic DOS and PDOS for the d electrons of Fe and Ti atoms and p electrons of O atoms for pristine $\;{\text{aTiO}}_{2}$, Fe(II)-$\;{\text{aTiO}}_{2}$, and rutile (1 1 0) surfaces. The DOS and PDOS are represented in Figure 2 for all the surfaces. It can be seen from Figure 2 that the magnitude of the Γ point electronic gap, defined as the difference between the highest occupied molecular orbital (HOMO) and the lowest unoccupied molecular orbital (LUMO), is about 2.2, 1.8, and 1.6 eV for pristine $\;{\text{aTiO}}_{2}$, Fe(II)-$\;{\text{aTiO}}_{2}$ and rutile (1 1 0) surfaces, respectively. Further, for all the surfaces the valence band mainly consists of the O 2p states and that the conduction band is dominated by Ti 3d states. In addition, the DOS analyses also reveals that there exist a midgap state for $\;{\text{aTiO}}_{2}$ surface due to O 2p orbitals (Figure 2(a)). However, it disappears when $\;{\text{aTiO}}_{2}$ is doped with Fe(II) (Figure 2 (b)). The midgap states can act as a recombination or a trapping center [24] and hence can decrease or increase the catalytic activity of $\;{\text{aTiO}}_{2}$ surface accordingly. The band tail states for Fe(II)-$\;{\text{aTiO}}_{2}$ surface however act as a trapping center and improve the photoactivity of Fe-$\;{\text{aTiO}}_{2}$ by absorbing light in visible region as reported in our previous study [24].

### Adsorption energy calculation for $\;{\text{H}}_{2}\;\text{O}$, OH and H species

3.2.

The adsorption energies of $\;{\text{H}}_{2}\;\text{O}$, OH and H species on the amorphous surfaces were calculated as

(1) $$\begin{array}{c} \delta H_{\;\text{ads}}=E_{\;\text{tot}}-E_{\;\text{bare}}-E_{\mathrm{ad}} \end{array}$$

where $E_{\;\text{tot}}$ ($E_{\;\text{bare}}$) is the energy of the surface with (without) adsorbate and $E_{\mathrm{ad}}$ is the energy of the isolated adsorbate species calculated in the same supercell. Hence, a negative $\delta H_{\;\text{ads}}$ indicates stable adsorption whereas a positive value indicates unstable adsorption. The author also calculated the adsorption of $\;{\text{H}}_{2}\;\text{O}$ molecule and dissociated $\;{\text{H}}_{2}\;\text{O}$ on rutile (1 1 0) surface for comparison. Adsorption energies for the most stable site for all the surfaces are represented in Table 1.

**Table 1.** Calculated structural parameters and adsorption energies of $\;{\text{H}}_{2}\;\text{O}$, OH, and H for the most stable site on pristine $\;{\text{aTiO}}_{2}$, Fe(II)-$\;{\text{aTiO}}_{2}$ and rutile (1 1 0) surfaces. $\delta H_{\;\text{ads}}$ (eV) represents the adsorption energy, h(Å) represents the vertical height of the $\;{\text{H}}_{2}\;\text{O}$, OH and H species from the nearest surface atom, $d_{O-H}$ represents the OH bond lengths for OH and $\;{\text{H}}_{2}\;\text{O}$ molecules and $\alpha_{\;\text{HOH}}$ represents the H–O–H angle for $\;{\text{H}}_{2}\;\text{O}$ molecule.

Most stable species	$H_{\;\text{ads}}$ (eV)	h(Å)	$d_{O-H}$	$\alpha_{\;\text{HOH}}$
Pristine $\;{\text{aTiO}}_{2}$ surface				
$\;{\text{H}}_{2}\;\text{O}$ (on site Ti-6c)	-1.072	3.519	1.055, 1.002	107.285
OH (on site Ti-4c)	-4.199	1.838	0.976	–
H (on site a)	-5.541	0.976	–	–
Fe(II)-$\;{\text{aTiO}}_{2}$ surface				
$\;{\text{H}}_{2}\;\text{O}$ (on site Fe-6c)	-0.793	2.263	0.9978, 0.9878	104.716
OH (on site Fe-6c)	-1.288	2.069	0.994	–
H (on site c)	-4.807	0.977	–	–
Rutile (1 1 0) surface				
H${}_{2}$O (on site Ti-5c)	-0.9378	2.212	0.979, 0.979	109.775
OH(on site Ti5c)-H (on bidentate O)	-8.503	1.822 (OH-surface), 0.973 (H-surface)	0.980 (O–H)	–

As a first step, author has investigated the possibility of $\;{\text{H}}_{2}\;\text{O}$, H and OH adsorption on the pristine $\;{\text{aTiO}}_{2}$ surface. For $\;{\text{H}}_{2}\;\text{O}$ and OH adsorption a total of three surface Ti atom sites based on their different coordination number were identified: fourfold coordinated Ti (Ti-4c), fivefold coordinated Ti (Ti-5c) and sixfold coordinated Ti (Ti-6c). For H adsorption, all the O atom sites neighbouring to these three surface Ti atoms were investigated. The three sites for $\;{\text{H}}_{2}\;\text{O}$ and OH adsorption and the ten sites for H adsorption are highlighted in Figure 3 in blue and yellow, respectively. While it is possible that the other adsorption sites exist on the $\;{\text{aTiO}}_{2}$ surface, the sites that author identified are enough to provide an understanding of the relative strength of binding and the spread of adsorption energies.

**Figure 4.**
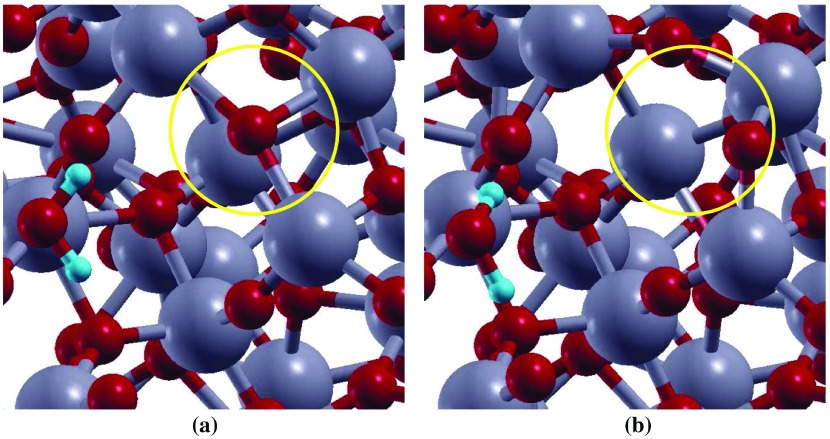
Top view representation of $\;{\text{H}}_{2}\;\text{O}$ adsorption on $\;{\text{aTiO}}_{2}$ surface (a) before, and (b) after optimization. Ti and O atoms are represented by grey, and red balls, respectively.

**Figure 5.**
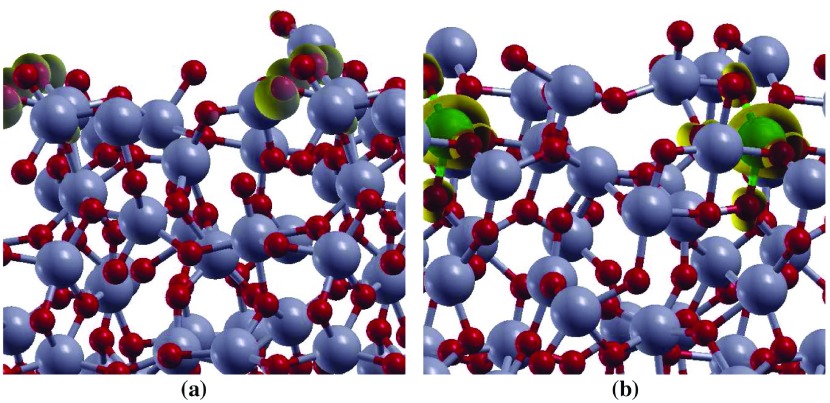
The spin density (ρ↑ – ρ↓) for (a) undoped $\;{\text{aTiO}}_{2}$, and (b) Fe(II)-$\;{\text{aTiO}}_{2}$ surface models. The isovalue used for spin density plots is 0.005 e${Å}^{-3}$. Ti, Fe, and O atoms are represented by grey, green and red balls, respectively. The spin density isosurface is represented in yellow color.

By analysing the interaction of $\;{\text{H}}_{2}\;\text{O}$ molecule on the three adsorption sites, surprisingly, it was found that binding of $\;{\text{H}}_{2}\;\text{O}$ with Ti-6c ($\delta H_{\;\text{ads}}=-1.07$ eV) is energetically preferred over under-coordinated Ti-5c ($\delta H_{\;\text{ads}}=-0.24$ eV) and Ti-4c sites ($\delta H_{\;\text{ads}}=-0.30$ eV). This result is contrary to the $\;{\text{H}}_{2}\;\text{O}$ adsorption on $\;{\text{cTiO}}_{2}$ surfaces [39]. On crystalline surfaces water prefers to adsorb on under-coordinated site. To explain this, $\;{\text{aTiO}}_{2}$ surfaces before and after water adsorption on all the sites were analysed. It was found that the atoms neighbouring to Ti-6c rearrange themselves after water adsorbs on this site (Figure 4). For instance, the O atom (circled) neighbouring to Ti-6c site and showing charge accumulation (represented by spin density diagram, Figure 5(a)) gets rearranged after water adsorption on Ti-6c site. However, such behaviour is absent when $\;{\text{H}}_{2}\;\text{O}$ is adsorbed on Ti-5c or Ti-4c site of $\;{\text{aTiO}}_{2}$ surface. This reconstruction of the surface due to the presence of water in the vicinity of Ti-6c site is probably the reason $\;{\text{H}}_{2}\;\text{O}$ is more stable on Ti-6c site compared to Ti-5c and Ti-4c site of $\;{\text{aTiO}}_{2}$ surface.

By comparing water adsorption on pristine $\;{\text{aTiO}}_{2}$ with water adsorption on rutile (1 1 0) surface (Table 1), it is clear that water is slightly more stable on sixfold coordinated Ti atom of $\;{\text{aTiO}}_{2}$ surface then on fivefold coordinated Ti atom of rutile (1 1 0) surface (Table 1), with a difference of about 0.13 eV in their adsorption energies. Further, the bond distance analysis shows that water adsorbs on the Ti-6c site with a 3.52 Å distance between the surface Ti atom and the O atom of water, which is higher than the existing experimental measurements (2.21±0.02 Å) [40] as well as our theoretical calculations for water adsorption on rutile (1 1 0) surface. The strongest adsorption energy and higher distance of water from the $\;{\text{aTiO}}_{2}$ surface indicate that $\;{\text{aTiO}}_{2}$ can be used to achieve highly stable anchoring of water contrary to crystalline surface on which water is comparatively less stable but chemically bonded. Furthermore, after dissociation of water on $\;{\text{aTiO}}_{2}$ surface, the most stable configration is the one in which the OH group binds to the fourfold coordinated Ti atom while the remaining H atom binds with the O atom coordinated to fourfold Ti. This indicates that even though the molecular adsorption of water is more probable at sixfold coordinated Ti atom, the dissociative adsorption of water might take place at under-coordinated surface Ti atom as on $\;{\text{cTiO}}_{2}$ catalyst.

In order to understand water dissociation on Fe(II)-$\;{\text{aTiO}}_{2}$ surface author then optimized three different configurations representing Fe(II)-$\;{\text{aTiO}}_{2}$ surface: Fe(II) doped at Ti-4c site (Fe(II)-4c-$\;{\text{aTiO}}_{2}$), Fe(II) doped at Ti-5c site (Fe(II)-5c-$\;{\text{aTiO}}_{2}$) and Fe(II) doped at Ti-6c site (Fe(II)-6c-$\;{\text{aTiO}}_{2}$) (Figure 3). In all these surface +2 oxidation state of Fe is achieved by substituting one Ti and one O atom with one Fe atom. Out of these three surfaces Fe(II)-6c-$\;{\text{aTiO}}_{2}$ was the most stable surface with total energy difference of -1.37 and -1.13 eV from Fe(II)-5c-$\;{\text{aTiO}}_{2}$ and Fe(II)-4c-$\;{\text{aTiO}}_{2}$ surfaces, respectively. Further, the spin density plot for the doped system (Figure 5(b)) shows that the spin densities are strongly localized on the Fe atom and O sites in the nearest-neighbour position relative to the Fe dopant.

Next, water adsorption on the most stable, Fe(II)-6c-$\;{\text{aTiO}}_{2}$, surface was analysed. The adsorption of $\;{\text{H}}_{2}\;\text{O}$ molecule was analysed by placing it on the top of Fe atom, resulting in the binding energy of -0.79 eV. Comparing with pristine $\;{\text{aTiO}}_{2}$ and rutile (1 1 0) surfaces, $\;{\text{H}}_{2}\;\text{O}$ is energetically least stable on Fe(II)-$\;{\text{aTiO}}_{2}$ surface. However, the distance (2.26 Å) between the Fe atom of the surface and the O atom of the water molecule is similar to the distance (2.21 Å) of adsorbed water on rutile (1 1 0) surface. Further, the author performed calculations for OH and H adsorption on Fe(II)-6c-$\;{\text{aTiO}}_{2}$ surface by placing OH on the top of Fe atom and H on top of four different O atom sites indicated by g, c, d and e in Figure 3. As for $\;{\text{H}}_{2}\;\text{O}$ adsorption, OH and H adsorption is also less stable when compared to OH and H adsorption on pristine $\;{\text{aTiO}}_{2}$ surface. The binding energy and vertical distance analysis indicate the Fe(II)-$\;{\text{aTiO}}_{2}$ surface might behave similarly to $\;{\text{cTiO}}_{2}$ surfaces for water adsorption and dissociation.

### 3.3. Reaction pathway for water dissociation

**Figure 6.**
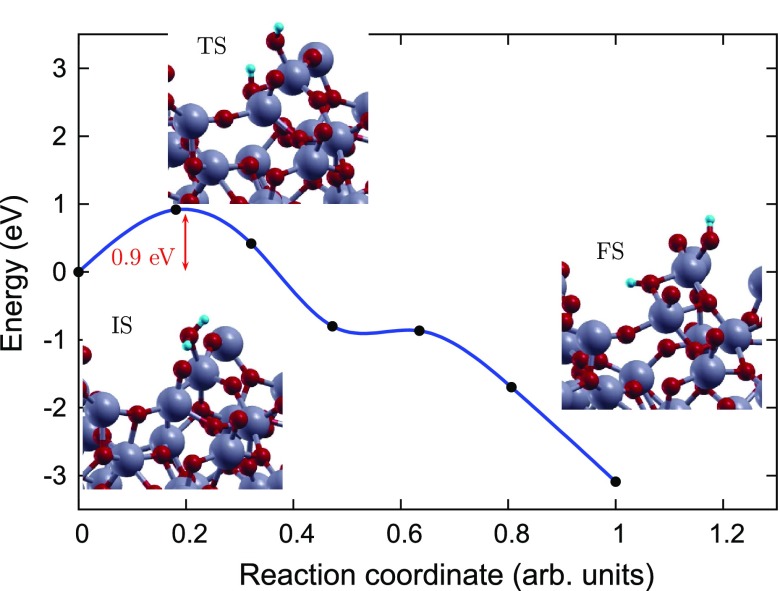
Reaction pathway and reaction barrier for dissociation of single water molecule on pristine $\;{\text{aTiO}}_{2}$ surface from CI-NEB simulation. Ti, O, and H atoms are highlighted in grey, red, and light blue, respectively.

**Figure 7.**
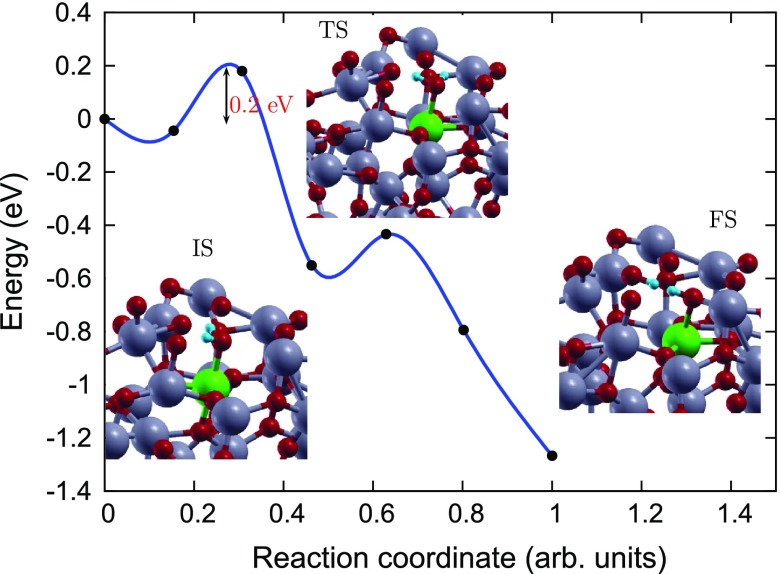
Reaction pathway and reaction barrier of single water molecule dissociation on Fe(II)-$\;{\text{aTiO}}_{2}$ surface from CI-NEB simulation. Ti, O, Fe and H atoms are highlighted in grey, red, green and light blue, respectively.

**Figure 8.**
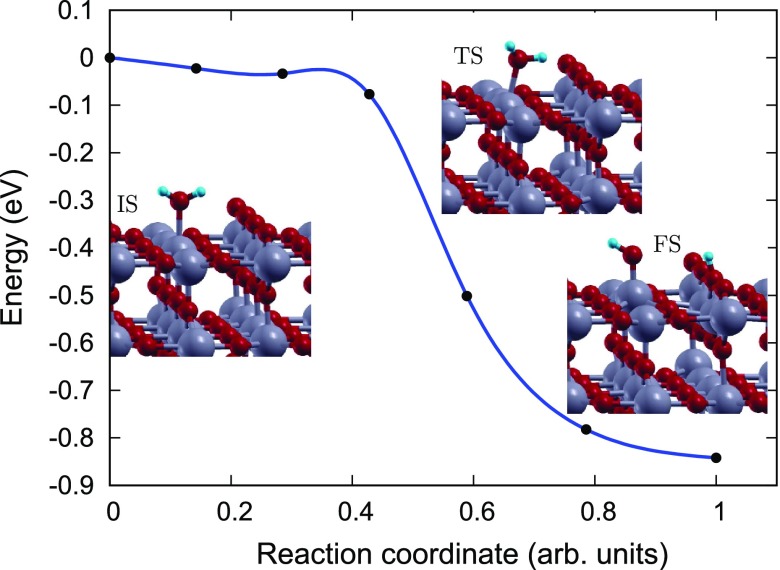
Reaction pathway and reaction barrier of single water molecule dissociation on rutile (1 1 0) surface from CI-NEB simulation. Ti, O, and H atoms are highlighted in grey, red, and light blue, respectively.

In order to gain an atomic scale understanding of the fundamental reaction pathway and get a sense of the relative activation energy barriers faced during water dissociation, CI-NEB calculations with 7 images were performed for all the surfaces. The preliminary results for the MEP and the TS energies for water dissociation on $\;{\text{aTiO}}_{2}$, Fe(II)-$\;{\text{aTiO}}_{2}$ and rutile (1 1 0) surfaces are represented in Figures 6–8, respectively, along with the initial state (IS), TS, and final state (FS) geometries. The chosen TS configration corresponds to the highest energy point along the MEP. The activation energy barrier, E${}_{a}$, is calculated as E${}_{a}$ = E${}_{\mathrm{TS}}$ – E${}_{\mathrm{IS}}$, and the reaction energy, ΔE is calculated as ΔE = E${}_{\mathrm{FS}}$ – E${}_{\mathrm{IS}}$, where E${}_{\mathrm{TS}}$ is the energy of the TS, E${}_{\mathrm{IS}}$ is the energy of IS and E${}_{\mathrm{FS}}$ is the energy of the FS. The negative ΔE indicates an exothermic reaction and a positive ΔE represents an endothermic one. Based on the most stable water adsorption sites (Table 1), Ti-4c, Ti-6c and Ti-5c sites were chosen for water dissociation on $\;{\text{aTiO}}_{2}$, Fe-$\;{\text{aTiO}}_{2}$ and rutile (1 1 0) surfaces, respectively. The IS corresponds to surface with chemically adsorbed $\;{\text{H}}_{2}\;\text{O}$ molecule, and the FS corresponds to the surfaces having dissociated water molecule with OH and H species adsorbed on the surface.

The reaction path was found to have similar TSs for both Fe(II)-$\;{\text{aTiO}}_{2}$ (Figure 7), and rutile (1 1 0) (Figure 8) surfaces [39], where the $\;{\text{H}}_{2}\;\text{O}$ molecule gets distorted resulting in elongated OH–H bond. However, for pristine $\;{\text{aTiO}}_{2}$ surface TS consists of dissociated $\;{\text{H}}_{2}\;\text{O}$ molecule with OH and H group bonded to the surface Ti and O atoms, respectively. Furthermore, our calculations of reaction energy, ΔE, indicate that water dissociation is exothermic on $\;{\text{aTiO}}_{2}$ (ΔE=-3.09 eV) and Fe(II)-$\;{\text{aTiO}}_{2}$ (ΔE=-1.27 eV) surfaces just like that on rutile (1 1 0) (ΔE=-0.84) surface and other $\;{\text{cTiO}}_{2}$ surfaces [41]. It should also be noted that, water splitting on Fe(II)-aTiO${}_{2}$ surface is less exothermic than on $\;{\text{aTiO}}_{2}$ surface and more exothermic than on $\;{\text{cTiO}}_{2}$ surfaces. The negative reaction energies for $\;{\text{aTiO}}_{2}$-based catalysts point towards favorable kinetics.

Further, the $\;{\text{H}}_{2}\;\text{O}$ dissociation is spontaneous on the rutile (1 1 0) surface but faces certain barrier for dissociation on amorphous surfaces. Activation energy barrier (E${}_{a}$) analyses shows that water dissociation on pristine $\;{\text{aTiO}}_{2}$ and Fe(II)-$\;{\text{aTiO}}_{2}$ surfaces has $E_{a}$ of 0.92 and 0.18 eV, making Fe(II)-$\;{\text{aTiO}}_{2}$ surface energetically more favourable for water splitting compared to pristine $\;{\text{aTiO}}_{2}$ surface. The smaller activation barrier for water dissociation on the Fe(II)-$\;{\text{aTiO}}_{2}$ surface is due to its unique band structure Figure 2. The tail states present in Fe(II)-$\;{\text{aTiO}}_{2}$ band structure (Figure 2(b)) might lead to better hybridization of energy states of the $\;{\text{H}}_{2}\;\text{O}$ molecule with the energy states of Fe(II)-$\;{\text{aTiO}}_{2}$ surface than the energy states of the pristine $\;{\text{aTiO}}_{2}$ surface. In other words, the existence of these resonating energy states in Fe(II)-$\;{\text{aTiO}}_{2}$ catalyst might make it easier for the H atom of the $\;{\text{H}}_{2}\;\text{O}$ molecule to bind with the neighbouring O atom of the surface upon $\;{\text{H}}_{2}\;\text{O}$ molecule interaction with the Fe-$\;{\text{aTiO}}_{2}$ surface.

## 4. Conclusions

In this study, DFT+U analysis was conducted for providing the mechanistic insights into the water splitting reaction on the $\;{\text{aTiO}}_{2}$-based catalysts. Our analysis showed that in contrast to crystalline surfaces $\;{\text{H}}_{2}\;\text{O}$ molecule tends to be more stable on sixfold coordinated Ti site then on under-coordinated sites of $\;{\text{aTiO}}_{2}$ surface. Further, the interaction between $\;{\text{H}}_{2}\;\text{O}$ and sixfold Ti site of $\;{\text{aTiO}}_{2}$ surface is stronger ($\delta H_{\;\text{ads}}=-1.07$ eV) than the interaction between $\;{\text{H}}_{2}\;\text{O}$ and fivefold Ti site of rutile (1 1 0) surface ($\delta H_{\;\text{ads}}=-0.94$ eV). The higher $\;{\text{H}}_{2}\;\text{O}$ stability on the pristine $\;{\text{aTiO}}_{2}$ surface could be attributed to the difference in the surface states of the adsorption sites where the spatial arrangement of the O atoms with reference to the Ti atom plays a major role. Furthermore, the density of states analyses and the CI-NEB analysis conducted in this study points towards better photocatalytic activity of doped $\;{\text{aTiO}}_{2}$ as compared to pristine $\;{\text{aTiO}}_{2}$ for water splitting. Overall, the present study reinforces the usefulness of doping in amorphous $\;{\text{TiO}}_{2}$ catalysts providing an alternative path to prepare an efficient and cost-effective visible light photocatalysts.
